# Design and Performance Evaluation of a Deep Neural Network for Spectrum Recognition of Underwater Targets

**DOI:** 10.1155/2020/8848507

**Published:** 2020-08-01

**Authors:** Dali Liu, Xuchen Zhao, Wenjing Cao, Wei Wang, Yi Lu

**Affiliations:** ^1^School of Electronics and Information Engineering, Tiangong University, Tianjin, China; ^2^State Key Laboratory of Acoustics, Institute of Acoustics, Chinese Academy of Sciences, Beijing, China; ^3^School of Electrical Engineering and Automation, Tiangong University, Tianjin, China; ^4^Tianjin Jinhang Computing Technology Research Institute, Tianjin, China

## Abstract

Due to the complexity of the underwater environment, underwater acoustic target recognition (UATR) has always been challenging. Although deep neural networks (DNN) have been used in UATR and some achievements have been made, the performance is not satisfactory when recognizing underwater targets with different Doppler shifts, signal-to-noise ratios (SNR), and interferences. In the paper, a one-dimensional convolutional neural network (1D-CNN) was proposed to recognize the line spectrums of Detection of Envelope Modulation on Noise (DEMON) spectrums of underwater target-radiated noise. Datasets of targets with different Doppler shifts, SNRs, and interferences were designed to evaluate the generalization performance of the proposed CNN. Experimental results show that compared with traditional multilayer perceptron (MLP) networks, the 1D-CNN model better performs in recognition of targets with different Doppler shifts and SNRs. The outstanding generalization ability of the proposed model shows that it is suitable for practical engineering applications.

## 1. Introduction

Underwater target recognition is one of the important functions of the sonar system, which plays a significant role in underwater situation perception. Due to the complex underwater environment, it is challenging for us to extract underwater target features. Therefore, underwater target recognition has always been a popular and challenging problem for sonar researchers [1].

Various sensors and signal processing methods are applied to extract features and identify underwater targets, such as Brillouin scattering [2], underwater optical imaging [3], and radiation noise feature extraction [4]. Based on these detection methods, some excellent projects used underwater interaction to raise awareness and access to European underwater cultural heritage [5]. For traditional underwater acoustic target recognition (UATR), the radiated noise of underwater targets and echoes reflected from these targets are the main signals applied to target recognition for passive sonar [6, 7] and active sonar [8, 9], respectively. With the advantages of long detection distance and strong concealment, the passive recognition mode based on target-radiated noise has attracted more attention from researchers.

The passive recognition method mainly uses traditional detection of envelope modulation on noise (DEMON) and low-frequency analysis and recording (LOFAR) to extract target features. The UATR widely uses the DEMON method because it can get the invariable physical characteristics of the target. Wang et al. used the high-speed characteristic quantity (HSCQ) of the DEMON spectrum to classify high-speed underwater targets [10]. Wu et al. combined the GT cepstral coefficient with the characteristic vector of DEMON spectrum analysis to improve the accuracy and stability of target classification effectively [11]. Yao and Hui combined the analytical acoustic intensity flux (AAIF) with the DEMON spectrum to propose a multitarget detection technique [12]. Although the abovementioned results have achieved the feature extraction task, these processes often require a large amount of human participation and are too dependent on subjective consciousness and experiences [13–15].

In the recent years, as the most popular deep learning model, the deep neural network (DNN) has attracted the interest of scholars in the field of UATR [16]. Yang et al. combined the auditory perception principle and convolutional neural network (CNN) to propose an auditory perception-inspired deep convolutional neural network (ADCNN), which used a CNN to extract features of different frequency components from signals and merged them at the fusion layer to achieve the classification of acoustic targets [17]. Choi et al. used the absolute values of matrix elements in the cross-spectrum density matrix (CSDM) to generate two additional matrices as input data and used them for training the CNN model [18]. Cao et al. combined the CNN architecture with a second-order pooling (SOP) and used a convolution layer to learn the local features of the data extracted by constant-Q transform (CQT), with achieving an end-to-end network to accomplish the classification of underwater targets [19]. Zhou et al. proposed a compound convolutional neural network based on the shared latent sparse (SLS) feature and deep belief network (DBN), using these two functions to learn fringe-based sonar images to improve the accuracy of classification [20]. Chen et al. proposed a method based on a convolutional neural network with residual units to recognize a time-frequency image of ship-radiated noise [21]. Wang et al. combined improved antinoise power-normalized cepstral coefficients (ia-PNCC) with a CNN and applied multitaper and normalized Gammatone filter banks to improve the antinoise capacity [22]. At the same time, the multilayer neural network and robust adaptive controller also optimized the target tracking control of the underdriven autonomous underwater vehicles (AUV), improving the anti-interference ability of underwater target recognition in the face of complex marine environments [23, 24]. Besides, multidimensional fusion networks [25] and the support vector machine (SVM) [26] are also used for UATR tasks.

Although the neural network has made some achievements in the field of radiated noise recognition of underwater targets, in actual engineering applications, there are still many problems that affect the recognition accuracy. Due to the relative movement between the underwater target and the sonar system, the sonar receives an underwater radiated noise containing the Doppler frequency shift. Also, due to the influence of the environment noise and the target distance, the signal-to-noise ratio (SNR) of the received radiated noise is not constant. Besides, because of environmental or human interferences, redundant spectrum lines may appear in the DEMON spectrum of the received radiated noise, or some spectrum lines may weaken or even disappear. When training a DNN, the training dataset cannot cover all the abovementioned situations. Though a trained DNN can recognize the targets in the dataset very well, it is challenging to recognize targets that are different from the training samples. As we all know, the acquisition of radiated noise data of underwater targets is costly. Therefore, this paper aims at improving the generalization ability of a DNN to identify the underwater targets with different Doppler shifts, SNRs, and interferences in the case of a limited dataset.

First, the model of underwater target-radiated noise was built, and then, the subband processing was performed to obtain the DEMON spectrums. The DEMON spectrums of different kinds of targets constituted the datasets to train and validate the DNN. The DEMON spectrums of radiated noise were composed of line spectrums and continuous spectrums, and line spectrums were used for target classification and recognition in the paper. Validation datasets with different Doppler shifts, SNRs, and interferences were designed to evaluate the generalization performance of the DNN. By adjusting the structure and parameters of DNN, a one-dimensional CNN (1D-CNN) was proposed for the recognition of the radiated noise of underwater targets. To further understand the performance of the designed network, a multilayer perceptron (MLP) network was designed to compare with it. By evaluating the performance of the designed networks, the effects of the Doppler shift, SNR, and interferences on the network performance were obtained. The proposed 1D-CNN can recognize the radiated noise of targets in different situations and has outstanding generalization ability.

## 2. Signal Processing and Samples Generation

### 2.1. DEMON Processing of Underwater Radiated Noise

The passive sonar mainly uses ship-radiated noise to detect and locate underwater targets. Because the SNR of the target-radiated noise is extremely low and the effective signal is completely masked in the noise, the received signal needs to be processed to improve the SNR firstly.

The power spectrums of the ship's radiated noise are mixed spectrums composed of line spectrums and continuous spectrums. This paper focuses online spectrums, which can reflect some periodic stable vibration sources on the target. The line spectrums consist of a series of discrete sinusoidal components with frequencies *f*_1_, *f*_2_,…, *f*_*M*_, and the signal with the single-frequency carrier can be expressed as

(1) $$\begin{array}{c} L\left(t\right)=\left[1+\sum_{i=1}^{M}p_{i}\sin\left(2\pi f_{i}t+\phi_{i}\right)\right]\cdot \cos\omega t, \end{array}$$

where *f*_*i*_*, p*_*i*_, and *ϕ*_*i*_ are the frequency, modulation factor, and random initial phase of the *i*-th line spectrum, respectively, *M* is the number of the sinusoidal components, and *ω* is the carrier frequency.

The signal model of the received ship-radiated noise can be written as

(2) $$\begin{array}{c} S\left(t\right)=L\left(t\right)+N\left(t\right), \end{array}$$

where *N*(*t*) is the Gaussian white noise and *L*(*t*) is the underwater environment noise.

DEMON spectrum analysis is one of the important methods to recognize ship-radiated noise, and it can show us the inherent physical characteristics of the target, including the axial frequency and leaf frequency of the propeller [27]. The different frequencies and amplitudes of line spectrums in DEMON spectrums are the main features to identify different underwater targets.

To simplify the analysis, *L*(*t*) is supposed to have only one component, which can be written as

(3) $$\begin{array}{c} L^{\prime }\left(t\right)=A\left(1+m\sin\Omega t\right)\cdot \cos\omega t, \end{array}$$

where *A* is the amplitude of the signal, *m* is the modulation factor, 0 < *m* < 1, and Ω is the frequency of the modulation signal.

To obtain the modulation signal sinΩ*t*, signal *L*′(*t*) is processed by absolute value demodulation; then,

(4) $$\begin{array}{c} \left|L^{\prime }\left(t\right)\right|=A\left(1+m\sin\Omega t\right)\cdot \left|\cos\omega t\right|,\\ =A\left(1+m\sin\Omega t\right)\cdot \frac{4}{\pi }\cdot \left(\frac{1}{2}+\frac{1}{3}\cos2\omega t-\frac{1}{3\cdot 5}\cos4\omega t+\frac{1}{5\cdot 7}\cos6\omega t+\cdots \right),\\ =\frac{2A}{\pi }+\frac{2A}{\pi }\cdot m\cdot \Omega t+\frac{4A}{3\pi }\cos2\omega t+\frac{2mA}{3\pi }\sin\left(\Omega +2\omega \right)t+\frac{2mA}{3\pi }\sin\left(\Omega -2\omega \right)t+\cdots . \end{array}$$

The signal |*L*′(*t*)| is mainly composed of a constant component, sinΩ*t*, and other high-frequency components related to the carrier frequency *ω*. Therefore, the modulation signal can be obtained after |*L*′(*t*)| is filtered.

The discrete modulation signal, *x*(*n*), is analyzed by Fourier transform, which can be written as

(5) $$\begin{array}{c} X\left(f\right)=\sum_{i=1}^{N}x\left(n\right)\cdot e^{-\left(j2\pi fn/N\right)}. \end{array}$$

Then, we obtain the DEMON spectrum of the radiated noise of underwater targets. As the carrier frequency *ω* is unknown, the received radiated noise should be processed by a series of band pass filters before DEMON processing to avoid losing part of feature spectrums [28, 29].

**Figure 1:**
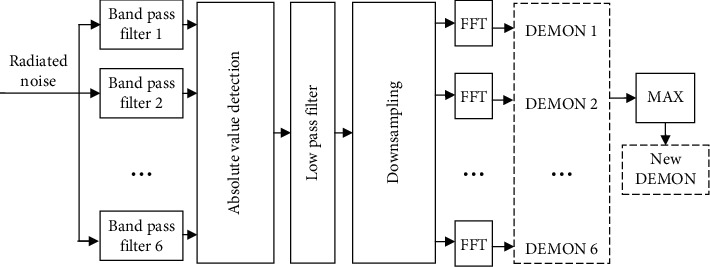
The proposed DEMON Processing.

In this paper, the radiated noise signal with the length of about 4.7 seconds and the sampling rate of 44.1 kHz was filtered by six band pass filters, and DEMON processing was applied to each obtained subband signals. The sampling rate of low pass filtered signals was reduced to 882 Hz before the Fourier transform, in order to reduce the calculation burden. Finally, the FFT length was set to 4096, and half of the FFT results constituted the DEMON spectrums with the length of 2048.  Figure 1 shows the proposed DEMON processing.

The *i*-th channel DEMON spectrum is *X*_*i*_(*k*), where 0 ≤ *i* ≤ 5 and *k* is the *k*-th value of discrete Fourier transform 0 ≤ *k* ≤ 2047. In order to further reduce the calculation of the DNN and retain the feature information as much as possible, for each frequency value *k*, the maximum value of the six channels was selected to compose a new DEMON spectrum, which can be described as

(6) $$\begin{array}{c} X^{\prime }\left(k\right)=\underset{i}{\max}X_{i}\left(k\right). \end{array}$$

The *X*_*i*_(*k*) can form a dataset with 6-channel samples that contain all the features of the underwater target, and *X*′(*k*) can form a single-channel dataset by data compression, which can reduce the time required for training and validating the DNN.

### 2.2. Datasets Generation

#### 2.2.1. Design of Training and Validation Datasets

To evaluate the effects of Doppler shift, SNR, and interferences on the performance of the DNN, the samples in the validation dataset need to contain all these parameters. It was not convenient to change these parameters in real underwater target-radiated noise, and it was not difficult to control the parameters in the simulated data described in equation (2) and generate different samples.

**Figure 2:**
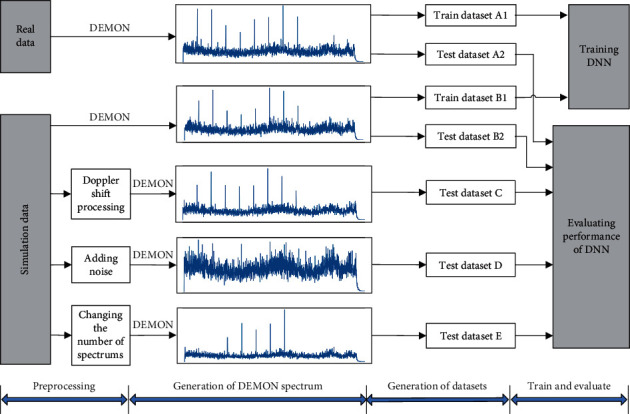
Design of training and validation datasets.

In this paper, seven kinds of targets were labeled 0 to 6, where targets 0–3 were real radiated noise, and targets 4–6 were simulated radiated noise. The DEMON spectrum of real target-radiated noise constituted dataset *A*, which was divided into dataset *A*1 and dataset *A*2. The two datasets were used for network training and performance evaluation, respectively. The simulated DEMON spectrum constituted dataset *B*, which was also divided into two parts: datasets *B*1 and *B*2, whose functions were the same as those of datasets *A*1 and *A*2. The validation datasets for evaluating the effects of Doppler shift, SNR, and interferences on the network performance were labeled as dataset *C*, *D*, and *E*, respectively.  Figure 2 shows the generation and function of all datasets.

#### 2.2.2. Generation of Validation Datasets

1. Generation of the dataset with different Doppler shifts (dataset *C*)
2. Due to the relative movement of the underwater target and the sonar platform, there will be a Doppler frequency shift in the received radiated noise of the underwater target. The sonar receives a radiated noise written as

(7) $$\begin{array}{c} L_{1}\left(t\right)=L\left(\eta t\right), \end{array}$$

  where *L*(*t*) is the target-radiated noise, *η* is the scaling factor of the Doppler effect, and *η* = *c*/(*c* + *v*)≈1 − (*v*/*c*) = 1 − *f*_*d*_; *v* is the relative radial velocity, *c* is sound speed in the water, and *f*_*d*_ = *v*/*c* is the relative Doppler frequency shift.
  By changing the value of *f*_*d*_, a series of target-radiated noises with different Doppler frequency shifts were generated, and samples of the validation dataset *C* were generated after DEMON spectrum processing. In this paper, the range of *f*_*d*_ was set from −0.02 to 0.02 in steps of 0.001 so that 41 kinds of validation samples with different Doppler frequency shifts were generated. In the training dataset *B*, the parameter *f*_*d*_ was set to zero to represent the data without frequency shift.
(2) Generation of the dataset with different SNRs (dataset *D*)
  Due to the influence of the underwater environment noise and the distance between the sonar and the target, the sonar received very different SNRs of the target-radiated noise that can be expressed as

(8) $$\begin{array}{c} L_{2}\left(t\right)=L\left(t\right)+\delta N\left(t\right), \end{array}$$

  where *N*(*t*) is Gaussian white noise, and *δ* is the coefficient that can control the noise power. By changing the value of *δ*, a series of target-radiated noises with different SNRs were generated, and samples of the validation dataset *D* were generated after DEMON spectrum processing. In this paper, the range of *δ* was set from 1.0 to 4.0, and the interval was 0.2 so that 16 kinds of validation samples with different SNRs were generated. Taking the power of the maximum line spectrum as the signal power, the SNR of the radiated noise was about 95 (19.8 dB) when *δ* = 1.0. In dataset *B*, *δ* was set to 1.0.
(3) Generation of the dataset with different interferences (dataset *E*)
  Due to environmental or human interferences, redundant line spectrums may appear in the DEMON spectrums of the radiated noise received by the sonar, or some line spectrums may weaken or even disappear. During the simulation, according to equation (1), by changing the number *M* of sinusoidal signals in *L*(*t*), a series of target-radiated noises with various interferences can be generated. Furthermore, samples of the validation dataset *E* were generated after DEMON spectrum processing. In this paper, the number of line spectrums was set from *M* − 3 to *M* + 4, so that eight kinds of validation samples with different interferences were generated. When the number of line spectrums is less than *M*, the line spectrums are lost. Otherwise, it means that there are interferences in the received radiated noise. In dataset *B*, the number of line spectrum was the standard value *M*, and there were no interferences and spectrum loss.

#### 2.2.3. Dimensions of the Training and Validation Datasets

We did actual surveys on four different ships to get four kinds of real underwater target-radiated noise in dataset *A* and 3000 samples for each kind. The DEMON spectrum of each sample was 2048 in size. Dataset *A* was split into training dataset *A*1 with 9600 samples and validation dataset *A*2 with the remaining 2400 samples, which means that all the samples were split into a 80% training dataset and 20% validation dataset. Dataset *B* contained three kinds of simulation targets whose frequency values of the line spectra were generated randomly within a reasonable range, and its split way was the same as that of dataset *A*. Each kind of target in datasets *A* and *B* had the same number of samples. Datasets *C*, *D*, and *E* also contained four kinds of real targets and three kinds of simulated targets, respectively, and the sample number of each kind was 500. For example, for a specific target in dataset *C*, the number of samples with relative Doppler shift 0.001 was 500. In order to evaluate the performance differences between the 6-channel DEMON spectrums and the single-channel DEMON spectrums, this paper designed a 6-channel dataset *X*1 and a single-channel dataset *X*2, respectively, and both datasets were composed of dataset *A*1, *A*2, *B*1, *B*2, *C*, *D,* and *E*. Tables 1 and 2 list the detailed information of datasets.

## 3. Design and Training of DNNs

### 3.1. Design of DNNs

There are many types of deep neural networks, and different networks are suitable for different types of tasks. We tried a few standard networks and finally chose CNN, a type of feedforward DNNs [30, 31] with a deep structure that includes convolutional calculations. It is one of the representative algorithms of deep learning, and CNNs have the capability of representational learning which can shift invariant classification of the input information according to its hierarchical structure. Therefore, it is also called “Shift-Invariant Artificial Neural Networks” (SIANN).

**Figure 3:**
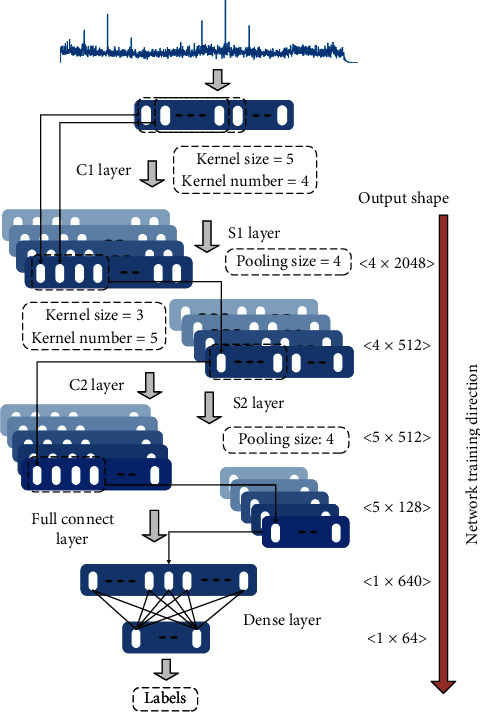
The architecture of the proposed 1D-CNN.

This paper proposed a one-dimensional CNN (1D-CNN) to recognize the DEMON spectrums of underwater target-radiated noise. It was expected that the network could have good generalization ability for the DEMON spectrums with different Doppler frequency shifts, SNRs, and interferences. Because there was no empirical information about the network structure, different parameters such as the layer number and kernel size were combined, and the CNNs with different combinations were trained, respectively. In this process, the number of layers was set from 2 to 5, the kernel size was set from 1 × 2 to 1 × 10, and the kernel number was set from 2 to 10. When the number of CNN layers exceeded three, its performance increased very slightly.  Figure 3 shows the optimal model of CNNs we have tried for the DEMON spectrums recognition. It consists of two convolutional layers, two pooling layers, a fully connected layer, and a hidden layer. The input of each neuron was connected to the output of the previous layer, which was used to extract local features [32, 33]. Four convolution kernels, each with the size of 5, were distributed in the convolution layer *C*1, whose input was a DEMON spectrum with 2048 elements. The output of layer *C*1 was four-channel data, and each channel also had 2048 elements. The pooling layer *S*1 was used for pooling data from the *C*1 layer, and the data were compressed into 512 so that this layer could use a smaller dimension to represent the feature data of the previous layer without losing valid information [34]. The *C*2 layer contained five convolution kernels, each with the size of 3; furthermore, its output was five-channel data, and each had 512 elements. When the data were pooled again by the *S*2 layer, the data length was reduced to 128. Then, the data were flattened and sent to the fully connected layer. Finally, after processed by the proposed deeply interconnected structure, the input samples were classified or identified.

The paper proposed two CNNs that were trained by a single-channel dataset and a 6-channel dataset, respectively. The only difference between the two CNNs is the size of the input layers. The input layer size of the CNN trained by a single-channel dataset is 1 × 2048 and the 6-channel dataset is 6 × 2048.  Figure 3 shows the structure of 1D-CNN trained by a single-channel dataset, and Table 3 lists the detailed information of the network parameters.

When evaluating network performance, in order to understand it more comprehensively, an MLP network [35] was designed to be compared with CNNs. Similarly, when selecting the MLP network parameters, the number of layers was set from 2 to 5 and the number of neurons was set from 32 to 2048 and was traversed various collocations for model selection. When the complexity of network reached a certain level, the increase in the number of layers was not helpful for performance improvement. Considering that a simple structure reduces the number of calculations for network propagation, the structural parameters of the MLP listed in Table 4 were finally selected.

### 3.2. Training of DNNs

Before training the networks, all network parameters were initialized by the Glorot initialization method that extracts samples from the truncated normal distribution with mean value *μ* = 0 and standard deviation$\sigma =\sqrt{2/\left(\text{fan\_in}+\text{fan\_out}\right)}$, where fan_in and fan_out are the numbers of input and output units of the weight tensors, respectively. For all convolutional layers, the rectified linear unit (RELU) was used as the activation function. During the training process, the dropout layer was added after the fully connected layer, and the dropout rate was set to 0.3 to avoid overfitting. One hundred and twenty-eight samples were extracted randomly from the dataset as a batch and input into the networks for training. The softmax layer converted the vector of 7 elements output by the last layer of the network into the probability distribution, which can determine which kind of target the input sample was. Back-propagation was performed by calculating the error between the predicted value and the real label, thereby updating the weights of the network. Finally, the training epoch was set to 15, and the parameters of the entire network were optimized by the Adam Optimization Algorithm [36]. The invariant structure information in a large number of training samples was extracted layer by layer in the network, and a method for underwater target recognition was realized.

## 4. Experiments and Discussion

### 4.1. Experimental Setup

The proposed CNN model was trained on the training datasets *A*1 and *B*1, and the performance of the model was evaluated on datasets *C*, *D*, and *E*. In order to understand the CNN performance more thoroughly, a carefully designed MLP network was compared with the CNN. This study focused on observing the changing trend of accuracy with different parameters, so it mainly evaluated the performance in the following three aspects:

1. When the received radiated noise contains a Doppler shift, is the proposed CNN trained by samples with no Doppler shift able to recognize the DEMON spectrums? Dataset *C* was used to evaluate the CNN and MLP networks for the recognition of targets with the Doppler shift.
2. When the SNR of the received radiated noise declines, can the proposed CNN trained by samples with high SNRs recognize the DEMON spectrums? Dataset *D* was used to evaluate the CNN and MLP networks for recognizing low-SNR targets.
3. When the received radiated noise contains interferences, can the proposed CNN trained by samples with no interferences recognize the DEMON spectrums? Dataset *E* was used to evaluate the CNN and MLP networks for recognizing targets with interferences.

Besides, the performances of two CNNs trained by 6-channel samples and signal-channel samples, respectively, were evaluated. Dataset *X*1 and *X*2 were used to evaluate the two CNNs about Doppler shift, SNR, and interferences.

### 4.2. Performance Evaluation of Doppler Shifts

**Figure 4:**
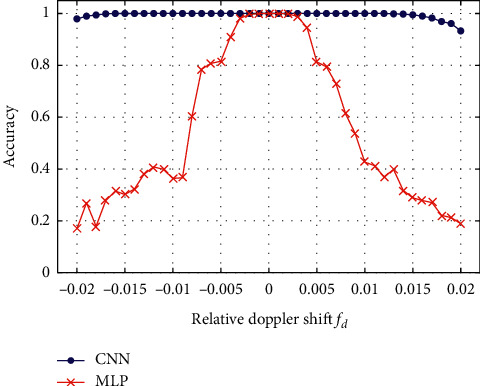
The performance of the proposed CNN and MLP networks for Doppler shifts.

Dataset *C* was used to evaluate the CNN trained by dataset *A*1 and *B*1 to understand the performance of the proposed CNN in recognizing targets with Doppler shift.  Figure 4 compares the evaluation results of CNN (blue line) with the performance of a well-designed MLP network (red line). The horizontal axis is the relative Doppler frequency shift, and the vertical axis is the accuracy of the networks for target recognition.

The figure highlights the high recognition accuracy of the MLP network when the relative Doppler shift is small (|*f*_*d*_| ≤ 0.003). However, as the Doppler shift increases, the accuracy declines rapidly. The results show that the MLP network is sensitive to the Doppler effect, and it is difficult for it to recognize the DEMON spectrums of underwater moving targets if trained by stationary samples. For the proposed CNN, the recognition accuracy remains almost constant when the relative Doppler shift is in a broad range (|*f*_*d*_| ≤ 0.015). When the relative Doppler shift increases to 0.02, the recognition accuracy slightly decreases and still maintains a value of more than 93%. The results show that the CNN has higher Doppler tolerance in DEMON spectrums recognition, and the proposed CNN trained by samples of stationary targets can still recognize fast-moving targets.

**Figure 5:**
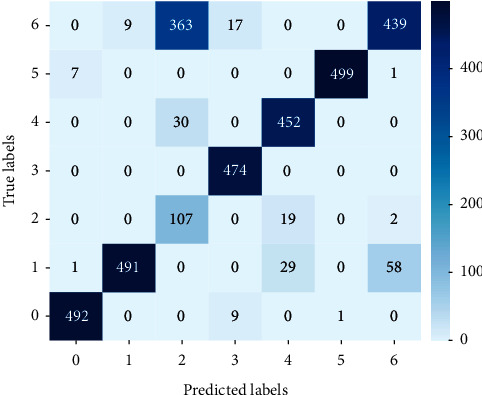
The classification results of samples with *f*_*d*_ = -0.02.

To further evaluate the recognition performance of CNN for dataset C, we analyzed the results of samples with the maximum relative Doppler frequency shift (*f*_*d*_ = −0.02), whose accuracy in Figure 4 was the worst. The classification results are shown in Figure 5, where the vertical axis is the actual label and the horizontal axis is the predicted label. There are 7 kinds of targets, and the sample number of each kind is 500. The color depth reflects the accuracy of recognition.

The precision and recall can be expressed as

(9) $$\begin{array}{c} {\text{precision}}_{k}=\frac{{\text{TP}}_{k}}{{\text{TP}}_{k}+{\text{FP}}_{k}},\\ {\text{recall}}_{k}=\frac{{\text{TP}}_{k}}{{\text{TP}}_{k}+{\text{FN}}_{k}}, \end{array}$$

where *k* is the sample label of the *k*-th kind, True Positive (TP) is correctly identified, False Positive (FP) is incorrectly identified, and False Negative (FN) is incorrectly rejected.

Therefore, each kind of *F*1-score can be written as

(10) $$\begin{array}{c} F1_{k}=\frac{2\cdot {\text{precision}}_{k}\cdot {\text{recall}}_{k}}{{\text{precision}}_{k}+{\text{recall}}_{k}}. \end{array}$$

By calculating the average value of the *F*1-score of each kind, *F*1_average_ can be written as

(11) $$\begin{array}{c} F1_{\text{average}}=\frac{1}{N}\sum_{k=0}^{N-1}F1_{k}, \end{array}$$

where *N* is 7.

For samples with the maximum relative Doppler frequency shift (*f*_*d*_ = −0.02), the calculation result of *F*1_average_ was 0.8256.

### 4.3. Performance Evaluation of SNRs

**Figure 6:**
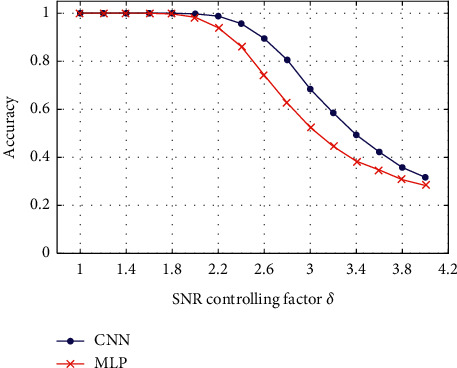
The performance of the proposed CNN and MLP networks for SNRs.

Due to underwater environment noise and the distance between the target and sonar system, the SNR of the received radiated noise is not constant. In order to understand the performance of the proposed CNN for recognizing targets with various SNRs, dataset *D* was used to evaluate the CNN and MLP network as shown in Figure 6. The horizontal axis is the value of *δ,* which can control the noise power, and the vertical axis is the accuracy of the networks.

According to the figure, both CNN and MLP networks can recognize targets well at high SNR values (*δ* ≤ 1.8). However, as the value of *δ* increases, the accuracies of CNN and MLP networks decline rapidly. The proposed CNN performs better than the MLP network, and the accuracy of the CNN is still higher than 80% when *δ* ≤ 2.8. The SNR is a crucial factor that can affect underwater target recognition for the DNN. If the SNR is in a reasonable range, the proposed CNN can recognize underwater targets with various SNRs.

**Figure 7:**
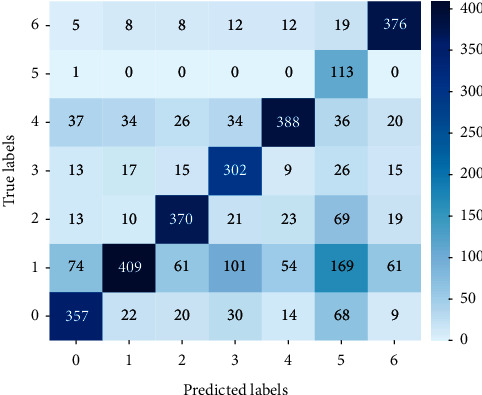
The classification results of samples with *δ* = 2.8.

We used the same method to obtain the classification results for the samples with the accuracy of 80% (*δ* = 2.8), as shown in Figure 7. Also, the calculation result of the *F*1_average_ was 0.6511.

### 4.4. Performance Evaluation of Interferences

**Figure 8:**
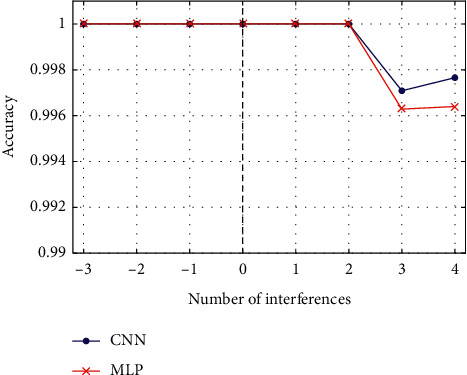
The performance of the proposed CNN and MLP networks for interferences.

Due to environmental or human interferences, redundant spectrums may appear in the DEMON spectrums of the received radiated noise, or some spectrums may weaken or even disappear. In order to understand the performance of the proposed CNN in recognizing targets with interferences, dataset *E* was used to evaluate the CNN and MLP network.  Figure 8 shows the results of the comparison; the horizontal axis is the number of interferences, and the vertical axis is the accuracy of the CNN and MLP networks. When the interferences are less than zero, spectrums with the same number are lost.

According to the figure, both the MLP network and the proposed CNN can recognize underwater targets with interferences well. A small disturbance of the number of feature spectrums little influences the performance of the DNN.

The recognition results were good enough, and further analysis was unnecessary.

### 4.5. Performance Evaluation of Channel Numbers

**Figure 9:**
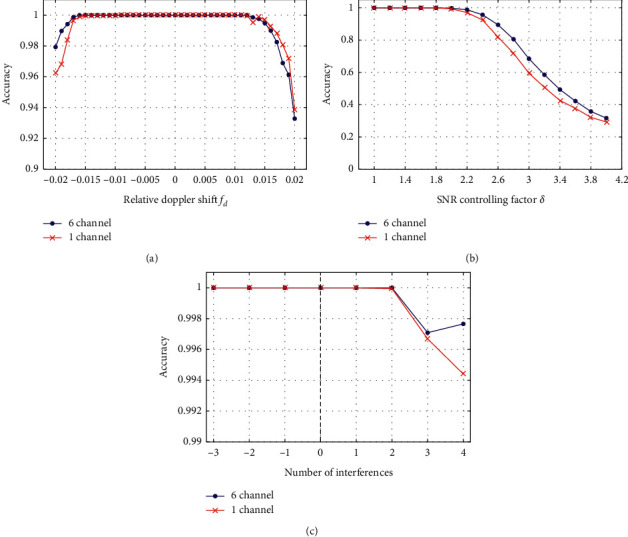
The performance comparisons of the 6-channel CNN and single-channel CNN. (a) Doppler shifts; (b) SNRs; and (c) interferences.

The received radiated noise was divided into six bands before absolute value demodulation order to obtain the DEMON spectrums with six channels. Then, the 6-channel samples were compressed into single-channel DEMON spectrums by selecting the maximum values to reduce the calculation burden. Dataset *X*1 of 6-channel samples and dataset *X*2 of single-channel samples were used to train and evaluate two CNNs; Figure 9 compares the performances.

In the figure, the CNN trained by 6-channel samples has slightly better accuracy than that trained by single-channel samples in the aspects of Doppler shift, SNR, and interferences. However, at the cost of a little accuracy loss, the total size of samples shrinks, which can reduce the training and validating time and storage of the CNN significantly.  Table 5 shows the training and validation time required for single-channel and multichannel datasets based on dual “NVIDIA GeForce GTX1080Ti” graphics cards. During the training process, the time required for the 6-channel CNN was 210.19 seconds and that of the single-channel CNN was 44.06 seconds. During the validating process, taking X1-C and X2-C for evaluating Doppler performance as an example, the time required for the 6-channel CNN was 158.58 seconds and that of the single-channel CNN was 31.03 seconds. The results show that the single-channel CNN can greatly reduce the calculation time for training and validation. Therefore, a trade-off should be made between the recognition accuracy and the available resources when recognizing DEMON spectrums of underwater radiated noise.

## 5. Conclusions

A one-dimensional CNN for recognizing the DEMON spectrums of underwater target-radiated noise was proposed, and the performances of recognition of targets with different Doppler shifts, SNRs, and interferences were evaluated. Compared with MLP networks, the proposed 1D-CNN has higher Doppler tolerance and can recognize underwater targets with lower SNRs. At the cost of a little accuracy loss, compared with the CNN trained by 6-channel samples, the CNN trained by single-channel samples can reduce the training and validating time and needs less storage. The simulation results show that the proposed 1D-CNN trained by single-channel samples has excellent performances in the DEMON spectrums recognition of underwater targets and is suitable for practical engineering applications.

## Figures and Tables

**Table 1:** The dimensions of the 6-channel dataset.

	Dataset	No. of events	No. of samples	No. of pixels	No. of channels	Data dimension
Training	*X*1*-A*1	1	9600	2048	6	9600 × 6 × 2048
	*X*1*-B*1	1	7200	2048	6	7200 × 6 × 2048

Validation	*X*1*-A*2	1	2400	2048	6	2400 × 6 × 2048
	*X*1*-B*2	1	1800	2048	6	1800 × 6 × 2048
	*X*1*-C*	41	3500	2048	6	41 × 3500 × 6 × 2048
	*X*1*-D*	16	3500	2048	6	16 × 3500 × 6 × 2048
	*X*1*-E*	8	3500	2048	6	8 × 3500 × 6 × 2048

**Table 2:** The dimensions of the single-channel dataset.

	Dataset	No. of events	No. of samples	No. of pixels	No. of channels	Data dimension
Training	*X*2*-A*1	1	9600	2048	1	9600 × 2048
	*X*2*-B*1	1	7200	2048	1	7200 × 2048

Validation	*X*2*-A*2	1	2400	2048	1	2400 × 2048
	*X*2*-B*2	1	1800	2048	1	1800 × 2048
	*X*2*-C*	41	3500	2048	1	41 × 3500 × 2048
	*X*2*-D*	16	3500	2048	1	16 × 3500 × 2048
	*X*2*-E*	8	3500	2048	1	8 × 3500 × 2048

**Table 3:** The structure and parameters of the proposed CNN.

Single-channel dataset CNN			
Layer type	Kernel number	Kernel size	Output shape
Input	—	—	1 × 2048
Conv_1D	4	1 × 5	4 × 2048
MaxPooling1D	1	1 × 4	4 × 512
Conv_1D	5	1 × 3	5 × 512
MaxPooling1D	1	1 × 4	5 × 128
Flatten	—	—	1 × 640
Dense	—	64	1 × 64
Output	—	7	1 × 7

**Table 4:** The structure and parameters of the proposed MLP.

Single-channel mode for MLP		
Layer type	Neuron size	Output shape
Input_layer	—	1 × 2048
Dense_1	1024	1 × 1024
Dense_2	256	1 × 256
Dropout_1	—	1 × 256
Dense_3	128	1 × 128
Dropout_2	—	1 × 128
Output_layer	7	1 × 7

**Table 5:** The time consumption of the single-channel CNN and 6-channel CNN.

	Type	Dataset	Time (s)
Training (20 epochs)	6-channel	*X*1*-A&X*1*-B*	210.19
	Single-channel	*X*2*-A&X*2*-B*	44.06

Validation	6-channel	*X*1*-C*	158.58
		*X*1*-D*	52.65
		*X*1*-E*	21.06
	Single-channel	*X*2*-C*	31.03
		*X*2*-D*	10.25
		*X*2*-E*	4.10

## Data Availability

The datasets used to support the findings of this study have not been made available because the datasets are too large. The datasets can be obtained from the author through e-mail.
